# Acute Cellular Rejection in Heart Transplant Patients: Insights of Global Longitudinal Strain, Myocardial Work, and an Exclusive Group of Chagas Disease

**DOI:** 10.3389/fcvm.2022.841698

**Published:** 2022-04-27

**Authors:** Maria Estefânia Bosco Otto, Aline Maria Araújo Martins, Aline de Oliveira Martins Campos Dall’Orto, Simone Ferreira Leite, Marco Antonio Freitas de Queiroz Mauricio Filho, Natalia Taveira Martins, Samuel Rabelo de Araújo, Soraya Vasconcelos Almeida, Mariana Ubaldo Barbosa Paiva, Fernando Antibas Atik

**Affiliations:** ^1^Cardiology and Transplant Heart Institute, Brasília, Brazil; ^2^School of Medicine, University of Brasilia, Brasília, Brazil

**Keywords:** heart transplantation, echocardiography, rejection, strain imaging, endomyocardial biopsy

## Abstract

**Background:**

Echocardiographic markers associated with asymptomatic acute cellular rejection (ACR) in patients with orthotopic heart transplant (HT) are still under investigation. The aim of our study was to determine clinical and myocardial strain imaging (MSI) variables evaluated by echocardiography associated with ACR in the first year of HT. A separate analysis was performed to compare variables during the first 6 months of HT, when ACR has a prevalence in 60% of patients. Another analysis evaluated an exclusive population with Chagas disease as the cause of HT.

**Methods:**

We prospectively studied 67 patients with less than 1 year of HT, 36 patients without ACR (41% men, age 49 ± 12 years, 52% Chagas disease as the cause of heart failure), and 31 patients with ACR (59% men, age 55 ± 8 years, 74% Chagas disease as the cause of heart failure). Conventional echocardiographic measurements and MSI by global longitudinal strain (GLS) from the left ventricle (LV) and right ventricle free wall (RV-FWLS) and myocardial work (MW) from the left ventricle were obtained by experienced echocardiologists. Clinical variables, such as the presence of diabetes, hypertension, and immunosuppressant drugs, were compared between groups.

**Results:**

HT patients with ACR were older and used more cyclosporine for immunosuppression. The positive ACR group had an increased relative wall thickness and LV mass index and similar LVGLS and RV-FWLS compared to the negative ACR group. Nevertheless, MW analysis observed increased global work efficiency (GWE) in positive ACR. Multivariate analysis identified older age, cyclosporine use, LV mass index, and GWE as independent predictors for detecting rejection. A separate analysis was performed for patients with less than 6 months of HT. Similar MSI was observed in both groups, with a trend for increased GWE in patients with ACR and significantly increased LV mass index in the ACR group. An exclusive group of Chagas patients as the primary cause of HT was analyzed, and similar MSI results for LVGLS, RV-FWLS, and MW were observed for both ACR and the no rejection groups. Additionally, the survival rates at 2 years were similar between the Chagas disease groups.

**Conclusion:**

LVGLS and RV-FWLS were similar between patients with or without ACR in the first year after HT. Conversely, GWE, a derivative of LVGLS, and LV mass index were increased in positive ACR and could be markers for rejection. Increased LV mass index was also found in a subgroup analysis of patients less than 6 months after HT; however, MSI was similar regardless of ACR. For chagasic patients, rejection in the first year did not increase mortality at the 2-year follow-up, and MSI parameters were similar between patients with or without ACR. In a multivariate analysis to predict ACR, the independent parameters in this study were older age, cyclosporine use, LV mass index, and GWE.

## Graphical Abstract

Illustrated abstract.

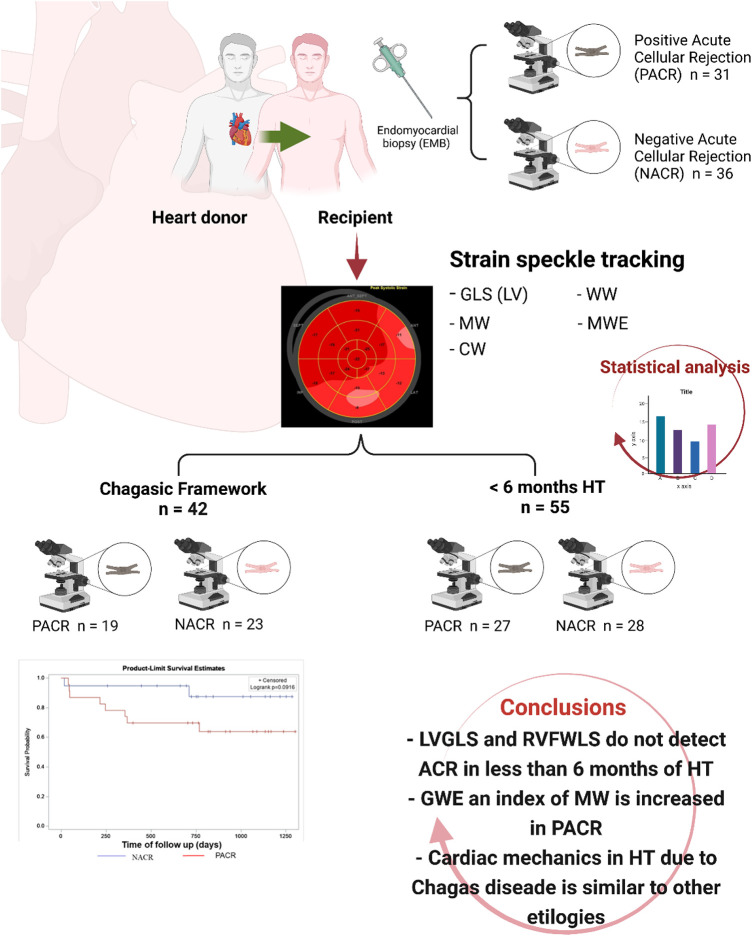

## Introduction

Heart transplant (HT) is the gold standard treatment for end-stage heart failure. Important improvements in patient selection and perioperative management have mitigated frequent postoperative complications, and early survival has improved dramatically. Nevertheless, acute cellular rejection (ACR) is a major problem in the early period after HT, and approximately 25–32% of patients experience some graft rejection in the first year (60% within the first 6 months) (1, 2). This is a major cause of death among patients with HT, particularly chagasic recipients in developing countries, occurring in 10–14% of all patients with ACR, despite efforts to develop new immunosuppressive protocols (1).

Most patients with ACR are asymptomatic or have non-specific symptoms, some degree of neurohormonal activation occurs (3), and endomyocardial biopsy (EMB) continues to be the best method for diagnosis (4). However, EMB is an invasive method with significant complications, including perforation, pneumothorax, cardiac tamponade, arrhythmias, and damage to the tricuspid valve (1). The pursuit of non-invasive alternatives to ACR has been a goal in the first year of HT (1, 5).

Asymptomatic ACR is not usually related to a reduction in left ventricular ejection fraction (LVEF). Nevertheless, new technologies in echocardiography by tissue Doppler analysis and global longitudinal strain (GLS) by speckle tracking are important to detect early myocardial injury despite normal LVEF (5).

A recent study by Mingo-Santos et al. (6) suggested that global LV and RV free wall longitudinal strain (FWLS) could help rule out significant ACR during the first year after HT. Other studies found similar results for GLS (7–9). Combined with biomarkers, Cruz et al. (7) reported that patients with ACR had significantly lower values of LVGLS, RV-FWLS, and LV-Twist and higher levels of troponin I than patients without significant ACR.

In contrast, Ambardekar et al. (10) found no changes in myocardial strain and strain rate as assessed by 2D-STE on serial studies from patients with asymptomatic biopsy-proven rejection in the first year after HT, agreeing with the findings of Tseng et al. (9).

Therefore, as a consequence of these conflicting findings regarding GLS in HT, echocardiographic markers for ACR are still under investigation. Furthermore, myocardial work (MW), an index derived from strain/pressure curves, has never been reported in HT (11).

Interestingly, despite HT, patients with Chagas disease still have other aspects of the disease, such as impairment of the autonomic nervous system (12), which could alter the neurohormonal response generated by ACR and Chagas reactivation as a differential diagnosis for ACR (1).

The aim of our study was to determine clinical and myocardial strain imaging (MSI) as evaluated by echocardiography associated with ACR in the first year of HT. We show an important methodological framework for the analysis of echocardiography in the first 6 months within an exclusive Chagas disease population as the cause of HT.

## Patients and Methods

### Patients

From January 2017 to December 2019, we prospectively included adult patients with less than 1 year of orthotopic HT at the Cardiology and Transplant Heart Institute, Brasília, Federal District, Brazil, in our study. Surveillance EMB was performed followed by a transthoracic echocardiogram on the same day, less than 4 h apart.

Patients were divided into two groups according to EMB results based on the 2004 International Society for Heart and Lung Transplantation (ISHLT) grading system (4): (1) without ACR (grades 0 and 1) and with ACR (grades 2 and 3). The HT group of rejection was compared to HT without rejection in terms of clinical and echocardiographic parameters.

### Ethics Statement

This study was approved by the Ethical Committee of Cardiology and Transplant Heart Institute of Federal District, Brasília, Brazil, and the inscription number in Plataforma Brazil as a Certificate of Presentation of Ethical Appreciation is 65910517.0.0000.0026. All patients provided written informed consent to participate in this study.

### Inclusion Criteria

We included patients with less than 1 year of orthotopic HT who came to our institution to undergo surveillance EMB and who were asymptomatic and hemodynamically stable. EMB and echocardiogram were performed on the same day. The protocol in our institution is to perform the echocardiogram less than 4 h after the EMB to diagnose any complications associated with the procedure (13) and before the discharge of the outpatients who only come for biopsy and follow-up.

### Exclusion Criteria

Patients with an ejection fraction below 53%, Chagas disease reactivation, humoral rejection (4), irregular cardiac rhythm, inconclusive EMB (1), and a limited echocardiographic acoustic window were excluded.

### Data Collection

**FIGURE 1:**
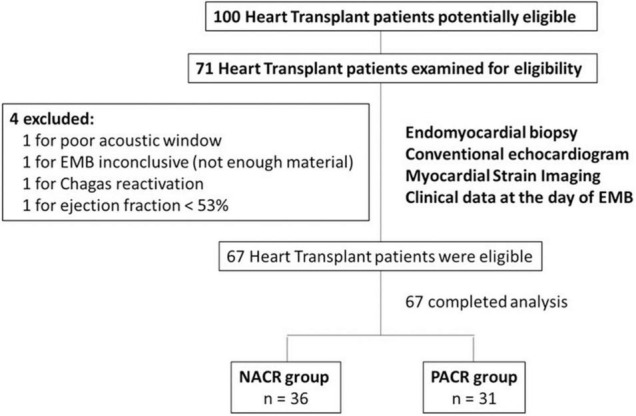
Flowchart of HT patients with or without acute cellular rejection. This study included 67 patients who underwent HT. Patients with poor acoustic window, with EMB inconclusive (not enough material), with Chagas reactivation, and with ejection fraction <53% were excluded. In the end, 67 HT patients were included in this study. Among them, 36 patients were in the NACR group and 31 patients were in the PACR group. Statistical analysis for sample size was performed with power analysis based on mean and standard deviation from a similar study (7). HT, heart transplant; EMB, endomyocardial biopsy; NACR, negative acute cellular rejection; PACR, positive acute cellular rejection.

Patients were enrolled at one routine follow-up EMB, only once within the first year of an orthotopic HT. In total, 71 patients were included, but 4 were excluded according to the exclusion criteria: 1 for poor acoustic window, 1 for inconclusive EMB (not enough material), 1 for Chagas reactivation, and 1 for ejection fraction <53%; 67 patients remained available for the study (Figure 1).

An endomyocardial biopsy was performed invasively through the femoral vein under fluoroscopy. Usually, a minimum of 3–5 segments were collected, aiming at the interventricular septal region, accessed by the right ventricle. The samples were analyzed by optical microscopy after hematoxylin and eosin (H&E) staining (1, 4). One pathologist blinded to the echocardiogram results analyzed all biopsies and classified cellular rejection according to the ISHLT grading system (4). Grades 0 and 1 are considered representative of not having significant cellular rejection (1). Grades 2 and 3 represent significant cellular rejection, and changes in immunosuppressor medications are usually necessary (1). EMB was performed according to our institutional protocol once a week in the first month of HT, every 15 days in the second month, and monthly from 3 to 7 months, for a total of 9 biopsies at 6 months. After 7 months of HT, cardiac scintigraphy is recommended when possible, and EBM is performed only when scintigraphy is positive for inflammation (1).

Echocardiography was performed by three trained cardiologists on a commercially available ultrasound machine equipped with a 5 MHz probe (GE Vivid 9, GE Healthcare, Milwaukee, WI, United States). Cardiologists were blinded to the biopsy results until all data were analyzed.

Standard echocardiogram images were acquired according to the recommendations of the American Society of Echocardiography (14). LVEF was obtained by biplane Simpson’s rule at apical 4- and 2-chamber views and LV mass was calculated using the equation proposed by Devereux et al. (14), indexed by body surface area. Relative wall thickness (RWT) was calculated as a ratio between 2 left ventricle posterior wall thickness and left ventricle end-diastolic diameter, with a value of >0.42 being considered abnormal (14). RV systolic function was assessed with conventional parameters recommended for routine clinical practice: tricuspid annular plane systolic excursion, systolic excursion velocity, and fractional area change obtained by M-mode, pulsed tissue Doppler, and two-dimensional echocardiography, respectively (14). Diastolic function was evaluated based on mitral inflow velocities (E and A), E/A ratio, annular mitral tissue Doppler velocities (e′/a′), and E/e′ ratio (15). All conventional and MSI analyses were performed offline on a workstation by the software EchoPAC Version 2.02 (GE Vingmed Ultrasound, Norway).

**FIGURE 2:**
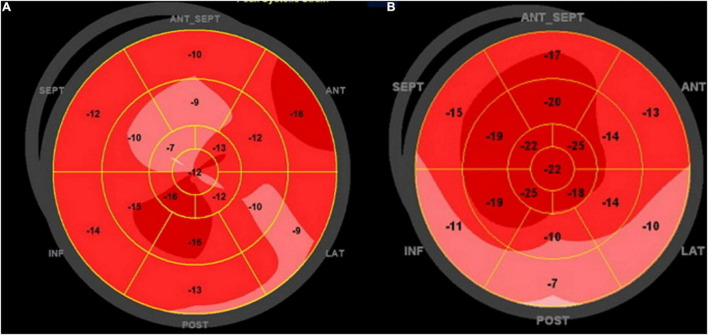
Global longitudinal strain polar map from 17 segments: **(A)** patient with NACR and **(B)** patient with PACR. Numbers inside each segment represent segmental strains color-coded in shades of red, and lower values are lighter shades. The strain was reduced in PACR and NACR with an irregular patch pattern as observed in the **(A,B)**.

**FIGURE 3:**
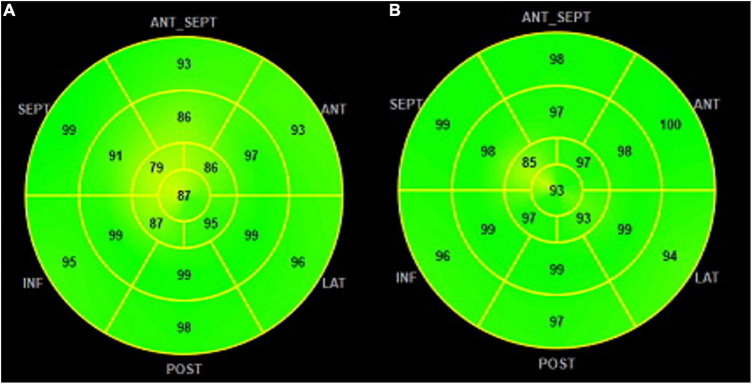
GWE polar map from 17 segments: **(A)** patients with NACR having a mean GWE of 96% and **(B)** patients with PACR having a mean GWE of 92%. Numbers inside each segment represent segmental work efficiency color-coded in shades of green, and lower values are in yellow shades. GWE was slightly higher in PACR compared to NACR. GWE, global work efficiency.

Left ventricle MSI was analyzed by GLS obtained by 2D speckle tracking (16). We obtained three consecutive heart cycles from each of the apical views (apical 4-chamber, 3-chamber, and 2-chamber), with frame rates above 50 per second. Endocardial borders were manually traced in the end-systolic frame of the cardiac cycle, starting from the apical long-axis view, where it is simpler to identify the timing of aortic valve closure. The software generated a region of interest (ROI) of the entire myocardial thickness, which could be manually adjusted in width if necessary, and a moving image displaying the tracking. If the tracking was considered inaccurate, the operator could repeat the process, readjust the ROI, or select a new ROI. The software then divided the left ventricle myocardium into six segments, calculating segmental and GLS. The same process was repeated for the apical 4- and 2-chamber images and the GLS was determined by averaging local strains of all myocardial segments and displayed in the format of a polar map (Figure 2). Using the same images for GLS of the LV and obtaining the systolic and diastolic blood pressures automatically during the image acquisition, we obtained the MW (11). The software used for MW analysis incorporates the left ventricular pressure non-invasively estimated through a cuff into the left ventricle strain, giving the indices associated with pressure-strain curves. Along with segmental and global values for the MW index, additional indices are provided:

• Constructive work (CW): work performed by myocardial segments during systolic shortening, which contributes to left ventricle ejection.
• Wasted work (WW): systolic lengthening during contraction (opposite from what is expected at the time of cardiac cycle) and does not contribute to left ventricle ejection.
• Global work efficiency (GWE) is the ratio CW/WW reported as a percentage (0–100%) (Figure 3).

The rationale for analyzing MW is that GLS has some dependency on loading conditions, making it difficult to distinguish between abnormal GLS due to intrinsically reduced LV contractility and increased LV afterload. MW can reduce the afterload-dependent limitation of GLS by incorporating blood pressure into the analysis (17).

Right ventricle MSI was analyzed by longitudinal strain from the RV-free wall. Totally, three cardiac cycles were obtained from the apical 4-chamber view focused on the right ventricle (14), and temporal markers of opening and closure of the pulmonary valve were obtained from continuous wave Doppler from the pulmonary artery at the level of the pulmonary valve. The average of the three right ventricular free wall segments, basal, medial, and apical, was considered the RV-FWLS.

The electronic medical records of all subjects were reviewed on the day of the EMB and echocardiographic studies to compare clinical, demographic, and drug intake for immunosuppressor treatment. The clinical characteristics collected were age, primary heart disease that led to HT, weight, height, body mass index, blood pressure, heart rate, hypertension, diabetes, and date of transplant to EMB. The list of immunosuppressant drugs included mycophenolate, cyclosporine, tacrolimus, azathioprine, and prednisone.

### Death Events Follow-Up for 2 Years

Patients with HT are routinely followed up at our hospital. Records of death events were followed prospectively for 2 years from the date of HT. The aim of this analysis was to check whether ACR could change mortality outcomes in the first year, mostly in chagasic patients, where autonomic dysfunction might be involved.

### Data Analysis

The calculated sample size for each group before data collection was based on other studies with HT and GLS analysis (7). The number obtained for each group was 34 subjects. With 34 patients in each group, the study would have a power of 80% to detect a clinically important reduction in the GLS of 10%, considering the normal value of 21%, standard deviation of 4%, and 5% alpha.

Continuous variables are presented as the means (SD), and categorical variables are presented as the numbers (percentage of total). Continuous data were analyzed using the Student’s *t*-test or the Mann–Whitney test when normality assumptions were not met. Categorical variables were analyzed using either the Pearson χ^2^ or Fisher’s exact test.

The multivariate analysis consisted of the adjustment of Poisson regression models with robust variance for a positive biopsy result associated with clinical and echocardiographic variables, using the prevalence ratio and its respective confidence intervals as an effective measure. Poisson regression was used because it provides a better estimate of the prevalence ratios and consequently more accurately represents the effect measures for cross-sectional studies. The analysis was performed in two stages: univariate and multivariate. In both, prevalence ratios and their respective 95% confidence intervals were calculated. In the univariate analysis, the association between each independent variable and the occurrence of a positive biopsy result was verified, and those with *p* < 0.25 were selected for inclusion in the multivariate analysis. In the multivariate analysis, the models were built by consecutively excluding a variable from each complete model that had the highest *p*-value in the Wald test and readjusting and verifying the stability of the model after the removal of each variable to best fit the data. Once the final model was obtained, the variables that had been excluded after the univariate analysis were added one by one to the model, and the Poisson regression analysis was repeated to identify the variables that could contribute to the model in the presence of other variables. Multicollinearity between independent variables was assessed. The limit for the presence of multicollinearity was a tolerance indicator of less than 0.40.

A framework study of patients with Chagas heart disease as the primary cause of HT was performed (23 with rejection and 19 without ACR) due to the high prevalence of this etiology in this prospective cohort (63% of all 67 patients), and the evaluation of mortality by a Kaplan–Meier curve within 2 years of follow-up was constructed to compare survival with the log-rank test in patients with and without rejection at the first year. Another framework group analyzed was HT patients with less than 6 months of follow-up, where the frequency of EMB was higher according to the protocol used for ACR control in our institution.

## Results

### Subsection A: Global Analysis

Patients were divided into two groups according to endomyocardial biopsy results: negative ACR (NACR) and positive ACR rejection (PACR). The clinical characteristics are described in Table 1. Patients with PACR were older (55 ± 8 vs. 49.3 ± 12 for NACR; *p* = 0.03) and were taking more cyclosporine (64 vs. 28% for NACR; *p* = 0.0026) and less tacrolimus (72 vs. 35% for NACR; *p* = 0.0026). A total of 80% (*n* = 54) of myocardial biopsies were performed in the first 6 months, and 20% (*n* = 13) were performed 6 months to 1 year from the date of transplant, probably related to the occurrence of a greater number of biopsies in the first 6 months, which was random. For primary heart disease, Chagas cardiomyopathy etiology was frequent but similar between groups (53% for NACR and 74% for PACR; *p* = 0.57).

**TABLE 1:** Clinical characteristics of heart transplant patients divided in NACR and PACR.

Characteristics	NACR (*n* = 36)	PACR (*n* = 31)	*p*-value
Age (years)	49.33 ± 12	55.19 ± 7.8	0.03
Gender female/male	25/11	15/16	0.08
SBP (mmHg)	129.17 ± 21	137.74 ± 26	0.14
DBP (mmHg)	83.67 ± 17	88.10 ± 16	0.29
HR (bpm)	84.78 ± 15	88.19 ± 13	0.32
Heigh (cm)	164.31 ± 7	163.90 ± 7	0.82
Weight (kg)	62.03 ± 10	65.51 ± 12	0.2
BSA (m^2^)	1.67 ± 0.2	1.71 ± 0.2	0.35
BMI (cm^2^/kg)	22.97 ± 3.6	24.40 ± 4	0.13
Time HT to EMB <6 m/>6 m	28/8	27/4	0.87
Hypertension			
**Diabetes**			
Primary heart disease			0.57
Chagas cardiomyopathy	19 (53%)	23 (74%)	
Idiopathic dilated cardiomyopathy	6 (18%)	4 (13%)	
Ischemic cardiomyopathy	3 (8%)	3 (10%)	
Valvular cardiomyopathy	2 (6%)	0	
Congenital cardiomyopathy	1 (3%)	0	
Postpartum cardiomyopathy	2 (6%)	0	
Non compaction cardiomyopathy	1 (3%)	11 (3%)	
CTRCD	1 (3%)	0	
**Imunossupressor drugs**			
Corticosteroids	34 (94%)	28 (90%)	0.52
Mycophenolate mofetil	25 (69%)	15 (48%)	0.08
Tacrolimus	26 (72%)	11 (35%)	0.003
Cyclosporine	10 (28%)	20 (64%)	0.003
Azathioprine	11 (30%)	16 (52%)	0.08
Time frame from HT to EMB (days)	89 ± 95	95 ± 98	0.52

*NACR, negative acute cellular rejection; PACR, positive acute cellular rejection; SBP, systolic blood pressure; DBP, diastolic blood pressure; HR, heart rate; BSA, body surface area; BMI, body mass index; HT, heart transplant; EMB, endomyocardial biopsy; CTRCD, cancer-therapeutic-related cardiac dysfunction.*

Analyzing conventional echocardiographic parameters, we observed higher RWT and left ventricle mass index in patients with PACR (0.5 ± 0.1 vs. 0.44 for NACR; *p* = 0.01 for relative wall thickness; and 96 ± 27 g/m^2^ vs. 89.4 ± 29 g/m^2^ for NACR; *p* = 0.045). Although E/e’ was lower in the PACR group (8.5 ± 4.6 vs. 11.9 ± 5.3 in NACR; *p* = 0.01), diastole could not be analyzed in more than 50% of patients due to technical problems, such as wave fusion (increased heart rate or abnormal atrial activation), making this result less reliable. All other echocardiographic conventional parameters were similar between groups and are displayed in Table 2.

**TABLE 2:** Conventional echocardiographic parameters.

Parameters	NACR (*n* = 36)	PACR (*n* = 31)	*p*-value
LVDD (mm)	10.03 ± 2.21	10.8 ± 2.09	0.16
PW (mm)	9.6 ± 1.84	10.6 ± 1.7	0.02
SW (mm)	43.7 ± 3.1	42.9 ± 4.9	0.48
Relative wall thickness	0.44 ± 0.1	0.50 ± 0.1	0.01
Ejection fraction (biplane Simpson) %	61.1 ± 8.8	64.5 ± 10	0.15
LV mass Devereux (g)	147.8 ± 43	162.0 ± 44	0.18
LV mass index (g/m^2^)	89.4 ± 29	96 ± 27	0.045
RV diameter (mm)	33.8 ± 6	35.2 ± 4.5	0.28
TAPSE (mm)	12.5 ± 3.2	12.2 ± 2.4	0.67
Lateral annulus tricuspid SV (cm/s)	7.4 ± 2.0	7.2 ± 2.2	0.71
RV FAC %	42.3 ± 6.9	44.8 ± 9.5	0.22
LA volume index (g/m^2^)	22.97 ± 3.6	24.40 ± 4	0.13
RA volume index (g/m^2^)	22.7 ± 10.2	24.04 ± 7.9	0.55
E velocity (cm/s)*	76.8 ± 20.7	72.4 ± 24	0.49
e′ septal (cm/s)*	6.7 ± 2.1	7.2 ± 1.8	0.33
e′ lateral (cm/s)*	8.7 ± 3.0	10.04 ± 3.3	0.11
E/e′*	11.9 ± 5.3	8.5 ± 4.6	0.01

NACR, negative acute cellular rejection; PACR, positive acute cellular rejection; LVDD, left ventricle diastolic diameter; PW, left ventricle posterior wall thickness; SW, septal wall thickness; LV, left ventricle; RV, right ventricle; TAPSE, tricuspid annular plane systolic excursion; FAC, fractional area change; LA, left atrial; RA, right atrial; E, mitral early systolic velocity; e′ septal, septal annulus early diastolic velocity; e′ lateral, lateral annulus early diastolic velocity. *Missing data due to technical limitations in 30% of NACR and 58% of PACR.

The MSI parameters are described in Table 3. All parameters of MSI were similar, except for GWE, which was higher in patients with PACR (89.1 ± 5% vs. 85 ± 8.7% for NACR; *p* = 0.03).

**TABLE 3:** Myocardial strain imaging parameters.

Parameters	NACR (*n* = 36)	PACR (*n* = 31)	*p*-value
LV GLS (absolute %)	12.1 ± 2.9	11.9 ± 2.7	0.83
RV FWLS (absolute %)	16.3 ± 5	16.5 ± 3.7	0.89
MWI (mmHg%)	1131.7 ± 469.4	1316.7 ± 508.2	0.12
GWE %	85 ± 8.7	89.13 ± 5	0.03
CW (mmHg%)	1395.9 ± 505	1541 ± 500	0.25
WW (mmHg%)	147.8 ± 43	162.0 ± 44	0.18

*NACR, negative acute cellular rejection; PACR, positive acute cellular rejection; LVGLS, left ventricle global longitudinal strain; RV-FWLS, right ventricular free-wall longitudinal strain; WI, myocardial work index; GWE, global work efficiency; CW, constructive work; WW, wasted work.*

A multivariate analysis was performed to assess variables that were independently associated with rejection, including clinical and echocardiographic characteristics. Based on the univariate analysis presented in Table 4, only the variables age, pretransplant diagnosis of myocardiopathy, BMI, systolic blood pressure, use of cyclosporine, end-diastolic posterior wall thickness, LV mass index, fraction area change of the right ventricle, and MWE were selected due to presenting *p* < 0.25 and were included in the complete model of the multivariate analysis. Applying this model, Table 5 shows the results for significant parameters in the adjusted prevalence ratio. The summary of these findings is described as follows: for every 1-year increase in age (1.04 years with a CI of 1.02–1.07 for PACR; adjusted prevalence ratio; *p* = 0.002), the prevalence of a positive biopsy result increased by 4%. Patients who used cyclosporine had a prevalence of positive biopsy results 82% higher than those who did not use cyclosporine (yes for cyclosporine 1.82 with a CI of 1.06–3.1; adjusted prevalence ratio *p* = 0.029). For every 1 g/m^2^ of indexed LV mass, the prevalence of a positive biopsy result increased by 1% (1 g/m^2^ with a CI of 1.00–1.01; adjusted prevalence ratio *p* = 0.04). For each increase of 1 GWE unit, the prevalence of a positive biopsy result increased by 5% (1.05% with a CI of 1.00–1.11; adjusted prevalence ratio *p* = 0.03).

**TABLE 4:** Univariate analysis for the presence of cellular rejection: tested parameters for gross prevalence ratios.

Parameters	GPR (CI)	*p*-value
Age (years)	1.03 (1.01–1.06)	0.0177
Cyclosporine use	–	0.029
No	1	–
Yes	2.18 (1.25–3.79)	0.0057
LV mass index g/m^2^	1 (1.00–1.01)	0.10
MWE %	1.05 (1.00–1.11)	0.0298
BMI Kg/m^2^	1.05 (0.99–1.02)	0.11
Hypertension	–	
No	1	
Yes	0.98 (0.51–1.88)	0.95
Diabetes		
No	1	
Yes	0.19 (0.03–1.22)	0.08
Lateral annulus tricuspid velocity (cm/s)	0.97 (0.85–1.10	0.66
TAPSE (mm)	0.98 (0.9–1.1)	0.71
RV FAC %	1.02 (0.99–1.05)	0.22
LV GLS% (absolute)	0.99 (0.9–1.1)	0.93
RV FWLS% (absolute)	0.99 (0.94–1.04)	0.67
RV FWLS% (absolute)	0.99 (0.94–1.04)	0.67

*GRP, gross prevalence ratio; LV, left ventricle; WE, myocardial work efficiency; BMI, body mass index; TAPSE, tricuspid annular plane systolic excursion; RV, right ventricle; FAC, fractional area change; LVGLS, left ventricle global longitudinal strain; RV-FWLS, right ventricular free wall longitudinal strain.*

**TABLE 5:** Multivariate analysis for the presence of cellular rejection.

Parameters	APR (CI)	*p*-value
Age (years)	1.04 (1.02–1.07)	0.0002
Cyclosporine use	–	0.029
No	1	–
Yes	1.82 (1.06–3.1)	0.029
LV mass index g/m^2^	1 (1.00; 1.01)	0.04
MWE %	1.05 (1.00; 1.11)	0.0298

*APR, adjusted prevalence ratio; CI, confidence intervals; LV, left ventricle; MWE, myocardial work efficiency.*

Interobserver variability was performed by comparing three different trained echocardiologist measures from 20 patients, and intraobserver variability was measured 1 month apart by one of the investigators. The intraclass correlation coefficient of LVGLS was 0.98 (95% CI = 0.94–0.99) for the interobserver variability coefficient and 0.88 (95% CI = 0.70–0.99) for intraobserver variability. The intraclass correlation coefficients of the RV-FWLS were 0.97 (95% CI = 0.94–0.95) for interobserver variability and 0.98 (95% CI = 0.95–0.99) for intraobserver variability. MW derived from GLS and the interobserver variability for each variable is described: GWI = 0.93 (95% CI = 0.84–0.97); GWE = 0.97 (95% CI = 0.93–0.99); GCW = 0.94 (95% CI = 0.85–0.97); and GWW = 0.92 (95% CI = 0.81–0.97).

### Subsection B: Chagas’ Study Framework

**FIGURE 4:**
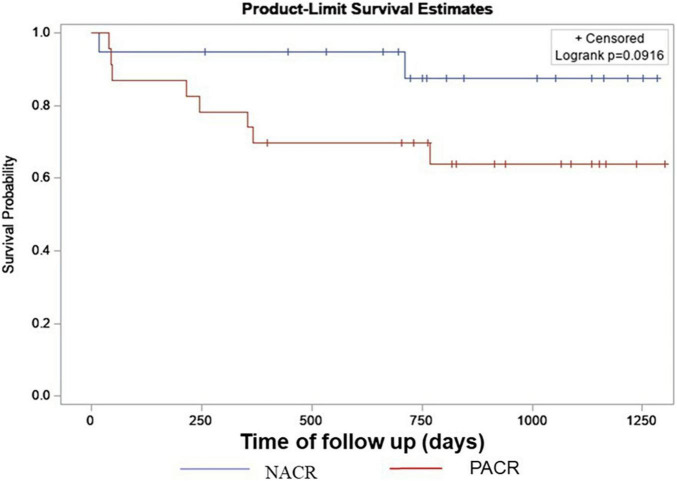
Kaplan–Meyer survival curve for Chagas with NACR and PACR. Results from a log-rank test of censored survival. NACR, negative acute cellular rejection; PACR, positive acute cellular rejection.

The most frequent cause of primary heart disease that led our patients to be subjected to HT was Chagas disease: 53% of NACR and 74% of PACR. To investigate the behavior of this important group in many developing countries, where Chagas is a frequent cause of heart failure, a framework analysis was performed to verify whether ACR increases mortality in 2 years where we had 8 deaths in PACR and 2 in NACR. A Kaplan–Meier survival curve was built to verify whether patients with prior Chagas cardiomyopathy and rejection had an increased risk of death (Figure 4). Patients in this group had a similar age between PACR and NACR (56 ± 8 years; 54 ± 3 years; *p* = 0.87, respectively). Although there was an apparent trend for worse survival for PACR, in the follow-up of 2 years, survival was similar in both groups (log-rank *p* = 0.09).

### Subsection C: Patients With Less Than 6 Months From HT to EMB Framework

A framework of patients with less than 6 months from HT to EMB was analyzed to verify whether echocardiographic parameters could discriminate NACR (28 patients) from PACR (27 patients) since a higher number of patients with less than 6 months was observed in this study (Table 7). Patients with PACR were older than those with NACR (49 ± 12 years and 55 ± 8 years, respectively; *p* = 0.035). Echocardiographic parameters were similar; only left ventricular mass index was higher in PACR, and GWE was borderline, with *p* = 0.05 for increased values in PACR (Table 6).

**TABLE 6:** Echocardiographic parameters for patients with Chagas heart disease as primary cardiomyopathy.

Parameters	NACR (*n* = 19)	PACR (*n* = 23)	*p*-value
LVDD (mm)	10.4 ± 2.1	10.5 ± 1.6	0.95
PW (mm)	9.79 ± 1.4	10.4 ± 1.6	0.20
SW (mm)	43.7 ± 3.1	43.7 ± 5.4	0.99
Relative wall thickness	0.45 ± 0.07	0.48 ± 0.08	0.19
Ejection fraction (biplane Simpson) %	59.8 ± 7	62.2 ± 9	0.54
LV mass index (g/m^2^)	92.8 ± 28.5	97.04 ± 29.6	0.64
RV diameter (mm)	33.5 ± 6.4	35.6 ± 3.5	0.20
TAPSE (mm)	11.9 ± 3.2	12.1 ± 2.5	0.91
Lateral annulus tricuspid SV (cm/s)	7.4 ± 2.2	7.2 ± 2.4	0.74
RV FAC %	42.3 ± 6.9	44.8 ± 9.5	0.22
LA volume index (g/m^2^)	37.8 ± 9	42.8 ± 10.5	0.12
RA volume index (g/m^2^)	24.3 ± 10.2	26.5 ± 7.2	0.31
LV GLS (absolute %)	11.4 ± 2.4	11.9 ± 2.7	0.49
RV FWLS % (absolute %)	16.5 ± 3.8	16.9 ± 6.8	0.76
WI (mmHg%)	1053.±421	1307.6 ± 532	0.1
MWE %	83.8 ± 10	88.8 ± 5.5	0.05
CW (mmHg%)	1373.8 ± 517	1522.8 ± 520	0.35
WW (mmHg%)	221.5 ± 181	137.6 ± 69	0.34

*NACR, negative acute cellular rejection; PACR, positive acute cellular rejection; LVDD, left ventricle diastolic diameter; PW, left ventricle posterior wall thickness; SW, septal wall thickness; LV, left ventricle; RV, right ventricle; TAPSE, tricuspid annular plane systolic excursion; FAC, fractional area change; LA, left atrial; RA, right atrial; E, mitral early systolic velocity; e’ septal, septal annulus early diastolic velocity; e’ lateral, lateral annulus early diastolic velocity; RV-FWLS, right ventricular free wall longitudinal strain; MWI, myocardial work index; MWE, myocardial work efficiency; CW, constructive work; WW, wasted work.*

**TABLE 7:** Echocardiographic parameters for patients with less than 6 months from heart transplant to EMB.

Parameters	NACR (*n* = 28)	PACR (*n* = 27)	*p*-value
LVDD (mm)	43.6 ± 23.4	42.1 ± 4.6	0.18
PW (mm)	9.9 ± 1.8	10.7 ± 1.7	0.09
SW (mm)	43.7 ± 3.1	43.7 ± 5.4	0.99
Relative wall thickness	0.46 ± 0.09	0.51 ± 0.09	0.03
Ejection fraction (biplane Simpson) %	62.1 ± 8.6	64.3 ± 10.9	0.44
LV mass index (g/m^2^)	89.3 ± 30.7	95.8 ± 29.1	0.044
RV diameter (mm)	34.5 ± 5.9	35.1 ± 4.8	0.69
TAPSE (mm)	12.4 ± 3.1	12.3 ± 2.5	0.80
Lateral annulus tricuspid SV (cm/s)	7.4 ± 2.2	7.2 ± 2.4	0.74
RV FAC %	43.6 ± 7.1	44.9 ± 9.6	0.53
LA volume index (g/m^2^)	43.4 ± 22.9	41.4 ± 10.7	0.68
RA volume index (g/m^2^)	24.0 ± 11.1	23.5 ± 7.8	0.83
LV GLS (absolute %)	11.9 ± 2.3	11.7 ± 2.5	0.73
RV FWLS % (absolute %)	15.3 ± 6.8	16.2 ± 5.6	0.61
MWI (mmHg%)	1073.9 ± 441	1313 ± 542	0.08
MWE %	84.7 ± 9	88.6 ± 4.9	0.05
CW (mmHg%)	1298.2 ± 410	1537.5 ± 534	0.07
WW (mmHg%)	174 ± 125	151 ± 66	0.91

*NACR, negative acute cellular rejection; PACR, positive acute cellular rejection; LVDD, left ventricle diastolic diameter; PW, left ventricle posterior wall thickness; SW, septal wall thickness; LV, left ventricle; RV, right ventricle; TAPSE, tricuspid annular plane systolic excursion; FAC, fractional area change; LA, left atrial; RA, right atrial; E, mitral early systolic velocity; e’ septal, septal annulus early diastolic velocity; e’ lateral, lateral annulus early diastolic velocity; RV-FWLS, right ventricular free wall longitudinal strain; WI, myocardial work index; MWE, myocardial work efficiency; CW, constructive work; WW, wasted work.*

## Discussion

The main findings of this study are that LVGLS or RV-FWLS values were not different in patients with less than 1 year of HT regardless of positive ACR. Conversely, GWE, a derivative of LVGLS, and LV mass index are increased in patients with ACR. Additionally, MSI parameters in patients with less than 6 months of HT are similar between groups of PACR and NACR. Other important findings are that echocardiographic parameters of Chagas cardiomyopathy as the primary cause of HT were not different in ACR, and the presence of rejection did not impose a higher risk of death in a follow-up of 2 years. Equally important, a multivariate analysis of the observed variables was able to determine independent parameters associated with ACR, such as older age, GWE, use of cyclosporine, and increased left ventricular mass index.

Endomyocardial biopsy is the gold standard for ACR. However, some complications associated with the method are not uncommon, such as myocardial perforation, pericardial tamponade, and iatrogenic tricuspid valve injury (1, 13), and 20% of patients have inconclusive biopsies related to sampling errors and variability in results due to pathological findings interpretation (18). Considering the limitations of EMB and the patient’s burden of undergoing nine biopsies in the first 6 months, non-invasive methods for the detection of ACR are of crucial interest.

Our study is the first to acknowledge MSI, conventional echocardiographic parameters, and clinical characteristics in the first year of HT. We highlight the analysis of specific groups, such as the first 6 months of HT, where ACR is reported in 60% of patients (2), and a population of primary Chagas disease as the cause of HT, where we observed that ACR at the first year in this group did not increase mortality in 2 years. In addition, for the first time, MW was described in HT, with some interesting results in GWE in patients with ACR.

Badano et al. (5) recommended the analysis of baseline HT echocardiogram 6 months after the procedure due to ischemic injury and residual right ventricular dysfunction and performed comparisons in subsequent exams to better diagnose ACR. Conversely, the first 6 months are the most important for preventing cellular rejection, making the analysis of parameters in this time frame relevant.

Our data contrast with earlier work showing decreased LVGLS and RV-FWLS in a population with more than 6 months of HT (7) or a smaller population with a lower rejection frequency with less than 1 year of HT (6). However, Tseng et al. (9) observed similar results for LVGLS in a retrospective study that included patients with less than 1 year of HT without considering the period of time between transplant and EMB of less than or more than 6 months. Ambardekar et al. (10) also found no changes in myocardial strain or strain rate as assessed by 2D-STE on serial studies from patients with asymptomatic biopsy-proven rejection in the first year after HT, with no mention of differences in the time frame of 6 months. In both studies, the GLS values were similar to those in this study. These conflicting findings in GLS and ACR might be related to the time frame of transplant and EMB.

Despite the superiority of GLS over LVEF in the deeper evaluation of LV systolic performance, this technique is still limited by loading dependence (11, 16), affecting the correct measurement of myocardial contractile function in specific loading conditions, such as higher blood pressure (17) or increased neurohormonal activation associated with HT rejection (3). In this context, MW analysis, incorporating LV pressure, is less load-dependent than strain and therefore could provide incremental information in the setting of patients with HT. To the best of our knowledge, this is the first study to analyze MW in patients with HT and observe that, compared to normal individuals (19), the indices of MW are diminished in both PACR and NACR subjects (Table 3). Nevertheless, GWE is significantly higher in PACR subjects than in NACR subjects, including 1-year HT and borderline HT, if only less than 6 months of HT are included. A possible hypothesis for this finding is that patients with PACR have increased afterload as a consequence of neurohormonal activation (3) and slightly higher blood pressure; to maintain normal ejection fraction, the contractile efficiency must be compensated (20). This finding of higher GWE in PACR is more evident after 6 months of HT since before 6 months, some degree of ischemic injury might be present in both groups (5) as a confounding factor for rejection injury. Interestingly, in an elegant study by Tokodi et al. (20), GLS was impaired in elite swimmers, while indices of MW were completely normal, maintaining normal LV function, probably as a consequence of a better description of myocardial contractility. In summary, MW indices mighty precociously differentiate PACR compared to GLS in the first year of HT.

In accordance with previous studies, our data observed ventricular remodeling in HT with increased LV mass index and RWT (21), predominantly in PACR. This was likely related to hypertrophy as well as edema caused by inflammation (5). RV parameters, such as FAC, tricuspid lateral annulus velocity, and TAPSE, were equally impaired in both groups, similar to the findings of Ingvarsson et al. (21).

In this study, the multivariable analysis identified independent predictors of ACR, such as age, predominant cyclosporine use as an immunosuppressor drug, LV mass index, and GWE. In contrast to previous studies, PACR occurred in older patients in this study. Normally, younger patients are more prone to ACR (22) as a consequence of a more active immune system (1). However, in our study, we observed an increased use of cyclosporine as a calcineurin inhibitor, which could be a confounding factor for the age variable, since older age could be associated with more cyclosporine utilization. Cyclosporine is known to be less effective than tacrolimus (23), making the presence of ACR more constant and possible statistical interaction of both variables, creating a bias in older subjects associated with increased ACR. In accordance with other studies, we confirmed an increased LV mass index and remodeling in PACR, likely associated with edema and hypertrophy in this group of patients (7, 21).

To the best of our knowledge, only one study has described MSI in HT with predominant chagasic cardiomyopathy as primary heart disease (7). Our study is the first to analyze a framework group with such characteristics and to observe similar conventional echocardiographic and MSI variables, both lower in PACR and NACR. In a Kaplan–Meier survival curve at the 2-year follow-up, the presence of ACR did not increase mortality in this group, despite possible impairment of the autonomic nervous system in these patients (12).

### Strengths of the Study

The strength of this study was to scrutinize the time frame of EMB and describe variables for ACR with less than 6 months of HT when the majority of ACR occurs. We were able to demonstrate decreased and similar values of MSI in both the PACR and NACR groups. Additionally, for the first time, MW was described in HT as a possible early marker for PACR compared to GLS to detect rejection using GWE in a court with 80% of patients with less than 6 months of HT. Finally, a separate analysis of chagasic cardiomyopathy was performed to describe survival in 2 years and possible changes in MSI.

### Limitations

The limitations of this study should be addressed. First, this was a single-center study with a small number of patients; nevertheless, a significant number of patients with ACR were enrolled compared to other studies. Another important comment is that GLS is an evolving method, and interobserver variability is high; thus, the values obtained in our study cannot be applied to other software for strain analysis. A multicenter study including other vendors and a higher number of patients must be performed. Our data could not determine the time frame where GLS is a marker for ACR; however, we were able to describe that MSI variables are not markers for ACR in a time frame of less than 6 months of HT.

## Conclusion

In the first year of HT, LVGLS, and RV-FWLS by speckle tracking were not able to detect changes in patients with asymptomatic ACR. Conversely, GWE, a derivative of LVGLS, and LV mass index are increased in patients with ACR and could represent possible markers for ACR. Increased LV mass index was also found in a subgroup analysis of patients less than 6 months after HT; however, MSI was similar regardless of ACR. In a subgroup analysis of chagasic patients, MSI was similar between groups with or without rejection, and ACR at the first year of HT did not increase mortality at the 2-year follow-up. In a multivariable analysis, parameters such as older age, use of cyclosporine, increased LV mass index, and increased GWE were independent variables related to ACR in this study.

## Data Availability Statement

The raw data supporting the conclusions of this article will be made available by the authors, without undue reservation.

## Ethics Statement

This research was approved by the Ethical Committee of Cardiology and Transplant Heart Institute, of Federal District, Brasília, Brazil and the inscription number in Plataforma Brazil as a Certificate of Presentation of Ethical Appreciation is 65910517.0.0000.0026. All patients signed an informed written consent to participate in this study. The patients/participants provided their written informed consent to participate in this study.

## Author Contributions

MO, FA, and AM were mainly responsible for the analysis of the data and manuscript writing. MO, AC, SL, and MQ were responsible for performing echocardiographic studies and MSI analysis. NM, SRA, SVA, and MP were responsible for data collection in medical records. MO and FA were responsible for the idealization of the study. All authors contributed to the article and approved the submitted version.

## Conflict of Interest

The authors declare that the research was conducted in the absence of any commercial or financial relationships that could be construed as a potential conflict of interest.

## Publisher’s Note

All claims expressed in this article are solely those of the authors and do not necessarily represent those of their affiliated organizations, or those of the publisher, the editors and the reviewers. Any product that may be evaluated in this article, or claim that may be made by its manufacturer, is not guaranteed or endorsed by the publisher.
